# Editorial: Understanding C_4_ Evolution and Function

**DOI:** 10.3389/fpls.2021.774818

**Published:** 2021-10-22

**Authors:** Martha Ludwig, Florian A. Busch, Roxana Khoshravesh, Sarah Covshoff

**Affiliations:** ^1^School of Molecular Sciences, University of Western Australia, Crawley, WA, Australia; ^2^School of Biosciences and Birmingham Institute of Forest Research, University of Birmingham, Birmingham, United Kingdom; ^3^Research School of Biology, Australian National University, Canberra, ACT, Australia; ^4^Department of Biology, University of New Mexico, Albuquerque, MN, United States; ^5^Independent Researcher, Las Vegas, NV, United States

**Keywords:** C_4_ photosynthesis, convergent evolution, comparative biology, biodiversity, regulatory mechanisms

C_4_ photosynthesis is a remarkable example of convergent evolution, having independently evolved at least 62 times over the last 60 million years (Sage et al., 2011). In C_4_ species, Rubisco operates close to its maximal carboxylation rate through suppression of the oxygenation reaction. This activity is accomplished via the establishment of a molecular CO_2_ pump that delivers carbon in the form of C_4_ acid intermediates to a spatially sequestered Rubisco. This carbon pump can be set up using a diverse array of complex biochemical and morphological modifications relative to the ancestral C_3_ photosynthetic state.

The large number of independent origins of a C_4_ syndrome suggests that evolution from ancestral C_3_ photosynthesis to a derived C_4_ type is flexible at the molecular level and relatively easy in genetic terms (Gowik et al., 2004; Williams et al., 2013; Heckmann, 2016). With a large pool of biodiversity to exploit, such as in Southwest Asia, reviewed here by Rudov et al., natural variation in diverse phylogenetic lineages can be used to better understand the molecular changes enabling evolution of a functional C_4_ syndrome. The papers presented in this Research Topic make use of this biodiversity to expand our knowledge of C_4_ evolution and function.

Despite C_4_ photosynthesis being highly convergent, little work has been done to understand which C_4_ traits have arisen through convergence and could be considered essential for a C_4_ syndrome. Here, Khoshravesh et al. use gas exchange, leaf ultrastructure and biochemistry and carbon isotope ratios to characterize the carbon assimilation pathways used by species in the eudicot family Nyctaginaceae, and in the case of the C_4_ members, to determine the subtype of C_4_ photosynthesis. Combining these data with those from other C_4_ clades, they compiled a hierarchical list of convergent and divergent traits.

Gene duplication has been proposed as one of the early steps in the recruitment of genes during evolution of a C_4_ pathway (Monson, 2003). Tronconi et al. describe a complex evolutionary history responsible for present-day C_4_-associated NAD-malic enzyme (NAD-ME) in the Brassicales that involves ancestral gene duplication followed by degeneration, complementation subfunctionalization, and neofunctionalization. Gene duplication and co-option also appear to be responsible for the evolution of the C_4_-associated PEP transporter, PPT1. Lyu et al. identify differences in coding and non-coding regions between C_3_ and C_4_ orthologs of PPT1 associated with increased expression of the transporter in C_4_ mesophyll cells (MC). They find that gene duplication and neo-functionalization led to recruitment of a PPT1 paralog found in roots to a role in C_4_ function.

Most C_4_ species operate a carbon pump with the help of Kranz anatomy, wherein MC surround highly specialized bundle sheath cells (BSC) that are concentrically arranged around the vasculature (Sage et al., 2014). In a small number of species, special organellar arrangements within a single cell are used to achieve high CO_2_ concentrations around Rubisco (Sharpe and Offermann, 2014). In work on *Suaeda aralocaspica*, a single-cell C_4_ species, Cao et al. identify paralogs encoding the C_4_-associated phosphoenolpyruvate carboxylase (PEPC), which catalyzes the first step in the C_4_ pathway, a housekeeping isoform, and a bacterial-type PEPC.

Given the apparent flexibility of gene recruitment during evolution of C_4_ syndromes, identification of regulatory components controlling the spatial expression of C_4_-associated enzymes is important for understanding C_4_ function. Here, Afamefule and Raines use C_3_ and C_4_ grasses to screen upstream regions of genes encoding four enzymes in the Calvin-Benson-Bassham (CBB) cycle for conserved nucleotide sequences that might enable cell-preferential expression. They identify *cis*-regulatory elements putatively involved in BSC-enriched expression of genes encoding CBB enzymes as well as candidate transcription factors potentially binding to those sites. In addition, Górska et al. identify three *trans*-acting factors that bind the upstream region of the C_4_-associated PEPC homolog of maize. Characterization of these factors highlights the complexity of cell-preferential expression in a C_4_ leaf and the role of repression in establishing some C_4_-type expression patterns.

Of course, evolution is ongoing. As suggested by the results of Moody et al. in a study on PEPCs from older and younger C_4_ lineages, optimization of the enzyme continues after a C_4_ syndrome is realized. Similar comparative studies of other enzymes in the C_4_ acid cycle may also contribute to our understanding of how a C_4_ syndrome evolves at the molecular level.

A better understanding of the molecular events underpinning evolution of a C_4_ syndrome could enable a C_3_ plant to be engineered for C_4_ features. This is highly desirable because C_4_ crops have higher yields and increased nitrogen and water use efficiency relative to C_3_ crops. Replicating the C_4_ process in C_3_ crops such as rice would therefore help feed a growing world population. Support for introducing a C_4_ pathway into rice is provided by Lin et al. Genes encoding four of the major enzymes in the maize NADP-ME-type C_4_ pathway, PEPC, NADP-malate dehydrogenase (NADP-MDH), NADP-ME and pyruvate phosphate dikinase (PPDK), were inserted into the rice genome. Subsequent measurements with ^13^CO_2_ demonstrate that production of ^13^C-labeled malate was high in the transformants, suggesting that a partial C_4_ pathway is functioning in these plants.

Studies on C_4_ physiology and metabolism are also important to improve breeding programs of C_4_ crops. In particular, light harvesting and nutrient availability and uptake are key determinants for crop productivity. Collison et al. explore relationships between leaf age and light availability with the phenomenon of shade maladaptation exhibited by the NADP-ME-type C_4_ crops maize, sorghum and sugarcane. Leaf age had little influence on the quantum yield of CO_2_ assimilation. Instead, optimization of the leaf light environment mitigates the negative effects on productivity associated with this maladaptive response. These results can inform breeding strategies related to canopy structure and agricultural practices such as planting densities to increase crop yield.

Jobe et al. highlight the need to consider the nutritional value of C_4_ crops in addition to yield. They review nutrient assimilation pathways in C_4_ plants and how they differ from C_3_ plants as well as discuss gaps in our knowledge of how nutrient uptake and levels are controlled in C_4_ plants. They also consider the effects of increasing atmospheric CO_2_ on C_3_ and C_4_ crop micronutrient assimilation and content in light of micronutrient-related malnutrition (i.e., hidden hunger). Such considerations are important for producing future C_4_ crops that will effectively address global food needs.

In summary, this collection of articles expands our understanding of C_4_ evolution and function. This new knowledge will inform future work in evolutionary biology, C_4_ metabolism, and crop improvement strategies.

## Author Contributions

All authors listed have made a substantial, direct, and intellectual contribution to the work and approved it for publication.

## Conflict of Interest

The authors declare that the research was conducted in the absence of any commercial or financial relationships that could be construed as a potential conflict of interest.

## Publisher's Note

All claims expressed in this article are solely those of the authors and do not necessarily represent those of their affiliated organizations, or those of the publisher, the editors and the reviewers. Any product that may be evaluated in this article, or claim that may be made by its manufacturer, is not guaranteed or endorsed by the publisher.
